# A Study of Using Massage Therapy Accompanied with Stretching Exercise for Rehabilitation of Mammary Gland Hyperplasia

**DOI:** 10.1155/2016/9426167

**Published:** 2016-02-28

**Authors:** Pin Lv, Yuping Chong, Huagang Zou, Xiangxian Chen

**Affiliations:** College of Physical Education, Anhui Normal University, Wuhu 241000, China

## Abstract

*Purpose*. To apply massage therapy accompanied with stretching exercises for treatment of mammary gland hyperplasia, evaluate the clinical outcome in patients, and estimate the therapy as a novel treatment method for mammary hyperplasia.* Methods*. 28 adult female patients were selected and treated with massage therapy and stretching exercises focusing on skeleton muscles of chest, abdomen, and axilla. The mammary gland oxyhemoglobin (OxyHb) and deoxyhemoglobin (DeoxyHb) levels were detected before and after treatment after 15, 30, and 45 days.* Results*. In this cohort, pretreatment OxyHb (mean ± SD) is 1.32 ± 0.14 (medium-high), and DeoxyHb is 0.87 ± 0.13 (normal). All patients were clinically diagnosed with benign mammary gland hyperplasia and mastitis. The posttreatment OxyHb levels are 1.23 ± 0.09 (normal-medium, 15-day), 1.16 ± 0.08 (normal, 30-day), and 1.05 ± 0.04 (normal, 45-day), and DeoxyHb levels are 0.90 ± 0.11 (normal, 15-day), 0.94 ± 0.18 (normal, 30-day), and 0.98 ± 0.12 (normal, 45-day). Patients were diagnosed with decreased hyperplasia 15 and 30 days after treatment and with no symptom of hyperplasia in mammary gland 45 days after treatment.* Conclusion*. Mammary gland hyperplasia is closely correlated with pathological changes of skeletal muscles and could be significantly improved by massage therapy and stretching exercises targeting neighboring skeletal muscles.

## 1. Introduction

Mammary gland hyperplasia (a.k.a. hyperplasia of breast) is the benign overgrowth of mammary ductal epithelial cells and interstitial fibers. Based on the developmental stages, mammary gland hyperplasia could be classified into mammary gland lobular hyperplasia, mazoplasia, cystic hyperplasia, and mammary dysplasia. Mammary gland hyperplasia is frequently diagnosed in adult females aged from 20 to 50 years, with an estimated rate of 68% in patients in Mainland China. The patients with mammary gland hyperplasia compose 99.3% of the total patients with breast-related diseases, with a 5–10% potential of developing breast cancer [1, 2]. Clinical symptoms of mammary gland hyperplasia include mastalgia, breast lump, pigmentation of nipples, and emotional swings, worsening during menstrual periods [3, 4]. It has been suggested that mammary gland hyperplasia was caused by the abnormal secretion of estrogen and progestogen during menstrual cycles, and patients were treated with steroid hormones. However, the hormone treatment caused little improvement in patients. On the contrary, sometimes it resulted in increased mastalgia, swelling, and interstitial fibrous nodules [5, 6]. In this study, female patients with benign mammary gland hyperplasia were treated with massage therapy and stretching exercise therapy focusing on the skeletal muscles on their chest, axilla, shoulder, and arms. Significant improvement of hyperplasia related condition was observed in patients, with diminished mammary gland lump, improved breast tenderness, and decreased pigmentation of nipples.

## 2. Materials and Methods

### 2.1. Patient Population

28 patients with benign mammary gland hyperplasia were selected after breast palpation, hemoglobin oxygen saturation analysis, and biopsy confirmation. All of their OxyHb levels were below 1.33 and DeoxyHb levels were over 0.87. The patients' ages range from 25 to 47, with an average of 38.4 ± 4.6. Their clinical history of mammary gland hyperplasia ranges from 3 months to 3.4 years, with an average of 1.8 ± 1.2 years. Among all the cases, 15 had mammary gland hyperplasia in both breasts, 6 in right breast only, and 7 in left breast only. The major symptoms of patients include breast and axillary lumps with various sizes, mastalgia, and nipple discharge.

Patients underwent optical imaging of breast by GJM breast oxygen functional imaging instrument [7] (Guoji Technologies Inc. (GJKJ), Cat # GJM—GT6). Images were acquired at multiple detective angles of mammary glands (excluding areola and nipple area) and processed for focal grey intensity remodeling, blood flow responses detection, grey level calibration, edge and blood vessel structure reconstruction, and so forth. OxyHb and DeoxyHb levels were measured and analyzed with the same equipment, with OxyHb level defined as normal (1–1.2), medium (suspicious) (1.2–1.4), and high (suspicious carcinoma) (>1.4) and DeoxyHb level defined as normal (0.9–1), medium (suspicious) (0.9–0.85), and high (suspicious carcinoma) (<0.85) [7, 8].

### 2.2. Massage Therapy, Exercise Prescription, and Rehabilitation Method

Every two days, patients got massage therapy one hour after meals in massage room. The therapy was carried out by four professional massage therapists. Within 24 hours after the therapy, the massaged areas were prohibited from exposing to water, wind, or cold air, and the patients were not allowed to eat cold meals. Every other day, patients performed stretching exercises, also carried out one hour after meals. The stretching practice took place in professional sports rehabilitation rooms and lasted for 30 minutes each time, with medium exercise intensity, and was always under the instruction of professional sports medicine therapists. 15, 30, and 45 days after starting of treatment, the mammary gland hemoglobin oxygen saturation status (OxyHb and DeoxyHb levels) was measured and analyzed.

### 2.3. Massage Prescription

Patients were lying flat, facing up or facing one side, relaxed with normal breathing rate. Therapists used their palms of hands, thumbs, and finger pads to rub, knead, push, and press to carry out the massage. The massaged areas include neck (subclavius muscle, sternocleidomastoid muscle, and scalene muscle), shoulder (trapezius muscle, pectoralis major muscle, and pectoralis minor muscle), abdomen (rectus abdominis muscle), axilla (serratus anterior muscle and latissimus dorsi muscle), and upper back (supraspinous muscle, infraspinous muscle, teres major muscle, and teres minor muscle). Each position was pressed and held for 8–10 times, at a frequency of 60–70 times per minute for 35 minutes. The massage therapy started at superficial layer of breast and aponeurosis with gentle strokes and friction movements and then got to deep tissues and went across the grain of the muscles with increased intensity (the maximum level within the comfortable range of each patient).

### 2.4. Stretching Exercises

*Targeted Muscles during Stretching*. They are triceps brachii muscle, teres major muscle, teres minor muscle, and serratus anterior muscle.

**Figure 1:**
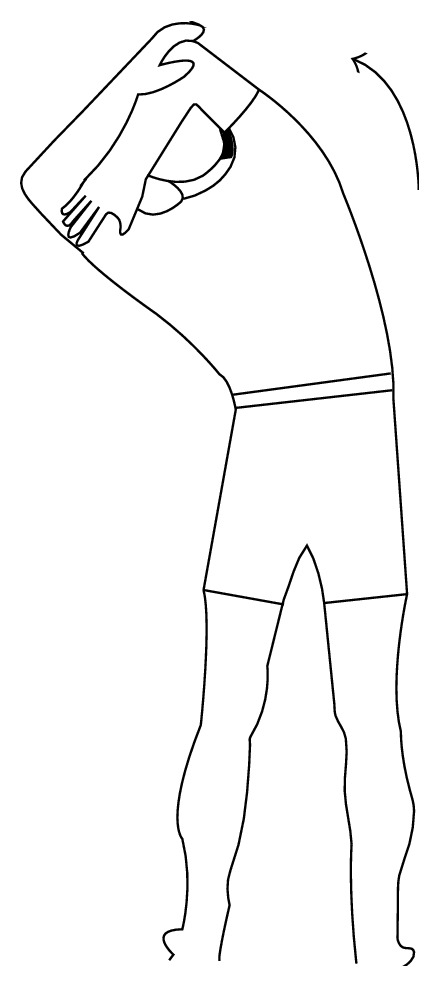


*Exercise 1*. Stand up straight, with both hands holding up at the back of neck and stretch to one side. Hold the position at the maximum angle for 30 seconds, and then slowly move back to straight. Repeat twice for both left and right sides (see Figure 1).

*Targeted Muscles during Stretching*. They are pectoralis major muscle, sternalis muscle, intercostal muscle, and rectus abdominis muscle.

**Figure 2:**
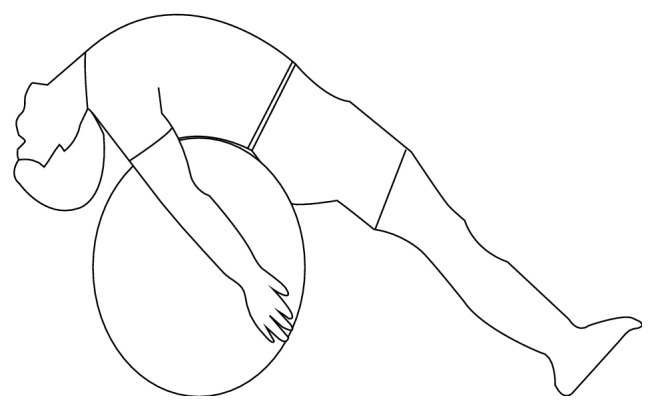


*Exercise 2*. Lie on the fitness ball with hands naturally down on side. Completely stretch the upper body and hold for 30 seconds. Repeat four times (see Figure 2).

*Targeted Muscles during Stretching*. They are pectoralis major muscle, pectoralis minor muscle, anterior deltoid muscle, and biceps brachii muscle.

**Figure 3:**
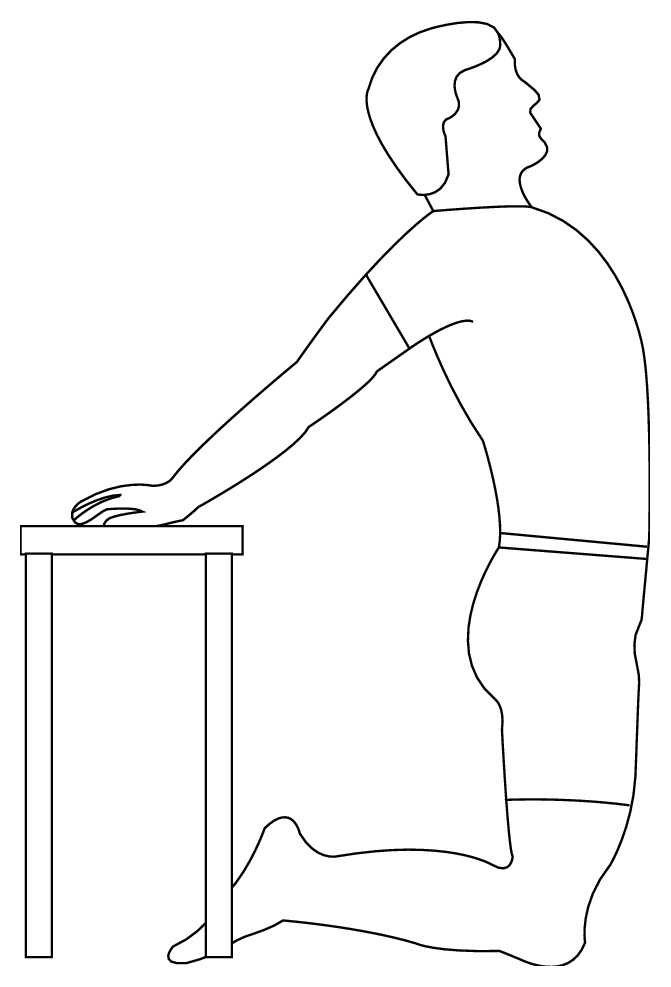


*Exercise 3*. Kneel down with back towards the table, with both hands stretched and placed on the table surface. Throw a chest to completely stretch the upper body, hold for 30 seconds, and slowly return to straight alignment. Repeat four times (see Figure 3).

*Targeted Muscles during Stretching*. They are pectoralis major muscle, pectoralis minor muscle, anterior deltoid muscle, and biceps brachii muscle.

**Figure 4:**
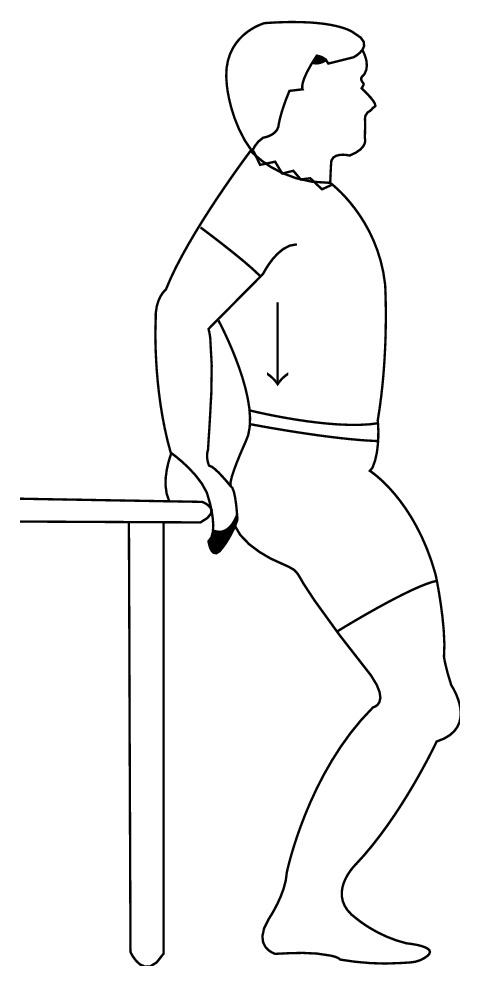


*Exercise 4*. Stand with back towards the table and hold both hands at the edge of table. Keep upper body straight and slowly bend knees until completely crouching down. Hold for 30 seconds and slowly stand up. Repeat four times (see Figure 4).

Image courtesy of Dr. Phil Page at Thera-Band Academy.

### 2.5. Statistical Analysis

The data were analyzed by SPSS for Windows version 17.0 (SPSS Inc., Chicago, IL, USA). One-way ANOVA analysis and Newman-Keuls test were used when appropriate (*p* < 0.05 was considered significant).

## 3. Results

In the patient group, pretreatment OxyHb (mean ± SD) is 1.32 ± 0.14 (medium-high), and that of DeoxyHb is 0.87 ± 0.13 (normal) (Table 1). All patients were clinically diagnosed with mammary gland hyperplasia and mastitis. At 15 days, the posttreatment OxyHb level is 1.23 ± 0.09 (normal-medium) and DeoxyHb level is 0.90 ± 0.11 (normal), with decreased hyperplasia (Table 1). At 30 days, the posttreatment OxyHb level is 1.16 ± 0.08 (normal) and DeoxyHb level is 0.94 ± 0.18 (normal), with significantly decreased hyperplasia (Table 1). At 45 days, the posttreatment OxyHb level is 1.05 ± 0.04 (normal) and DeoxyHb level is 0.98 ± 0.12 (normal), with no symptom of hyperplasia in mammary gland (Table 1). In this study we used massage therapy together with stretching exercises to treat the injured skeletal muscles closely located to mammary gland. This treatment method is supposed to relieve muscle spasm, accelerate blood flow, and increase oxygen capacity in muscles. It would also promote the lymphatic circulation for removal of algogenic and inflammatory substances, suppress the cell overgrowth of muscle fibrosis, soften knots in interstitial fibers in mammary gland, and decrease the tension in mammary gland. With this unique treatment method, the OxyHb and DeoxyHb levels in patients' mammary gland decreased from medium OxyHb, medium DeoxyHb pretreatment to normal OxyHb, normal DeoxyHb posttreatment, and the clinical conditions switched from mammary gland hyperplasia and mastitis to normal.

## 4. Discussion

Skeletal muscle injuries are one of the most common traumas (10–55%) in orthopedic and sports medicine, with the occurrence ranging from 10 to 55% [9]. This kind of injuries could result in symptoms such as pain, inflammation, muscular dysfunctions, and muscular dystrophy. The muscle regeneration after the injuries is often time consuming and incomplete (compromised by development of fibrosis), which will result in scar tissue formation and is more susceptible to reinjury [10–12]. In modern world, people often experience tension, spasm, and stiffness of muscles on chest, abdomen, and underarm areas, largely due to the common gestures used for reading, computer operating, or cell phone holding, with head lowered, chest shrank, shoulders rounded, and arms elevated. These gestures were maintained by the simultaneous contraction of sternocleidomastoid muscle, scalene muscle, subclavius muscle, pectoralis major muscle, pectoralis minor muscle, and rectus abdominis muscle. In adult females, breasts are commonly located between the second ribs and the sixth ribs, with inner margin close to sternum and outer margin close to anterior axillary line (sometimes to middle axillary line in patients with larger breasts). The mammary gland is more extensive than the breast and generally extends into the axilla as an “axillary tail” [13, 14]. Since breasts are located on the areas neighboring to chest, axilla, and upper abdomen areas, they cover on complete pectoralis major muscle, pectoralis minor muscle, sternalis muscle, and intercostal muscle and are close to rectus abdominis muscle and serratus anterior muscle. Long-time concentric contractions would cause muscle fatigue or injuries, leading to abnormal ultrastructure of myocytes and histiocytes, as well as changes in cellular metabolism and histochemical features in mammary gland. These pathological changes include decreased active oxygen, elevated free calcium in cytoplasm, induced hypoxia, and cell membrane dysfunction (such as occurrence of abnormal substances or abnormal accumulation of certain substances in cells and extracellular matrix). This would further affect the two major circulation systems involved in muscle functions, the cardiovascular system (for transportation of nutrient and oxygen, carbon dioxide, etc.) and the lymphatic system (for transportation of T- and B-cells), and would impair the normal signal transmission by nervous tissues [15]. In addition, this could also explain the phenotypes of pain, swollenness, and regional overgrowth related to mammary gland hyperplasia.

Based on these facts, we want to use massage therapy on injured skeletal muscles to treat related mammary gland diseases. According to feedbacks from patients, the massage on the deep tissues (but not the superficial layer) would cause the pain similar to the level of mastalgia they had experienced with mammary gland hyperplasia, indicating that the pain might also come from skeletal muscle tissues in addition to mammary gland tissues. The push-and-press techniques of massage therapy could help improve the congestion, effusion, and edema from injured skeletal muscle tissues, separate muscle adhesion, effectively decrease the tension in muscles and hamstrings and the irritation of transmitting nerves, and relieve the pain. The regular kneading of mammary gland will warm up the massaged area, accelerate blood circulation, and dredge lymphatic ducts on chest and axilla and inside breasts.

The parenchyma in mammary gland is arranged in about 15 to 20 lobes. Each lobe is drained by a lactiferous duct opening on the nipple, with all the ducts radiating from the center (nipple). Alternative pressing and releasing on the mammary gland function like a force pump, flushing away waste products and debris of cells together with bacteria and protein in clavicular and axillary lymph nodes [16]. The removal of algogenic and inflammatory substances will repress muscular fibroplasia and fibrosis, soften knots in interstitial fibers in mammary gland, and facilitate the blood delivery of nutrients into previously blocked regions [17].

The skeletal muscle injuries could not only induce mammary gland diseases, but also enhance the negative impacts related to gestures such as forward head, round shoulders, and shrank chests. Our stretching exercises focus on pectoralis major muscle, pectoralis minor muscle, serratus anterior muscle, deltoid muscle, sternocleidomastoid muscle, teres major muscle, teres minor muscle, and infraspinous muscle. In addition to restore extensibility and elasticity of skeletal muscles and to consolidate the results from massage therapy, the exercises could also help adjust the postures of patient and keep the patient's body in alignment. Another thing to mention is that the shortening of pectoralis major and pectoralis minor muscles could induce the dislocation of sternoclavicular joint, acromioclavicular joint, and glenohumeral joint, as those two muscles connect and largely overlap with those three joints [18]. When muscle is back to normal length, the pressure on chest is relieved, which improved the symptoms of palpitation, chest tightness, and dyspnoea in a few patients. Effective stretching exercises emphasize the strength of stretching, holding time, and frequency. To completely recover the elasticity of muscular fibers, the holding time for each stretching position is recommended to be a maximum of 30 seconds, so that tendon (the elastic tissues) could be extended thoroughly [19]. Studies showed that tendons and ligaments around the joints are composed of compact fibrous connective tissues (elastic and plastic collagenous fibers). Since collagenous fibers are oriented in parallel, spiral, or crossing patterns following tension lines, those tissues lack flexibility [20]. Consistent, slow, and repeated stretching could extend elastic fibers and stabilize the length of muscles. Short and fast stretching would only extend plastic fibers.

## 5. Conclusion

Breast tissues locate on chest, close to upper abdomen and axilla. The injuries of skeletal muscles behind breast could affect blood circulation, lymphatic metabolism, and nervous transmission in breast. In this study we used massage therapy together with stretching exercises to treat the injured skeletal muscles closely located to mammary gland. This treatment method is supposed to relieve muscle spasm, accelerate blood flow, and increase oxygen capacity in muscles. It would also promote the lymphatic circulation for removal of algogenic and inflammatory substances, suppress the cell overgrowth of muscle fibrosis, soften knots in interstitial fibers in mammary gland, and decrease the tension in mammary gland. With this unique treatment method, the OxyHb and DeoxyHb levels in patients' mammary gland decreased from medium OxyHb, medium DeoxyHb pretreatment to normal OxyHb, normal DeoxyHb posttreatment, and the clinical conditions switched from mammary gland hyperplasia and mastitis to normal. This strongly supports the idea that the incurrence of mammary gland hyperplasia is closely related to pathological changes of skeletal muscles and that we could target skeletal muscles for effective treatment of mammary gland hyperplasia. Besides, the massage therapy and stretching exercise therapy are simple, beneficial for self-care of breast, with no requirement of specific equipment or site. Based on the results from this study, it will be interesting to further explore the effect of massage therapy in assisting the treatment of common postmastectomy complications, such as lymphedema, scar contracture, and disabilities of arm, shoulder, and hand (DASH).

## Figures and Tables

**Table 1:** Mammary gland oxy/deoxyhemoglobin examination results (mean ± SD, *n* = 28).

Test time	OxyHb	DeoxyHb	Mammary gland Oxy/DeoxyHb level	Clinical diagnosis
Pretreatment	1.32 ± 0.14	0.87 ± 0.13	Medium OxyHb, medium DeoxyHb	Mammary gland hyperplasia and mastitis
Posttreatment				
15 d	1.23 ± 0.09	0.90 ± 0.11	Normal-medium OxyHb, normal DeoxyHb	Mammary gland hyperplasia and mastitis
30 d^*∗*^	1.16 ± 0.08	0.94 ± 0.18	Normal OxyHb, normal DeoxyHb	Mammary gland hyperplasia
45 d^*∗*^	1.05 ± 0.04	0.98 ± 0.12	Normal OxyHb, normal DeoxyHb	Normal

^*∗*^At 15 days, the difference between pre- and posttreatment levels of OxyHb and DeoxyHb in patients is not significant (*p* > 0.05). At 30 and 45 days, there is significant difference between pre- and posttreatment levels of OxyHb and DeoxyHb in patients (*p* < 0.05).
